# Clearance of *Streptococcus suis* in Stomach Contents of Differently Fed Growing Pigs

**DOI:** 10.3390/pathogens5030056

**Published:** 2016-08-06

**Authors:** Franziska Warneboldt, Saara J. Sander, Andreas Beineke, Peter Valentin-Weigand, Josef Kamphues, Christoph Georg Baums

**Affiliations:** 1 Institute for Animal Nutrition, University of Veterinary Medicine Hannover, 30173 Hannover, Germany; Franziska.Koop@googlemail.com (F.W.); saara.sander@tiho-hannover.de (S.J.S.); josef.kamphues@tiho-hannover.de (J.K.); 2 Department of Pathology, University of Veterinary Medicine Hannover, 30559 Hannover, Germany; andreas.beineke@tiho-hannover.de; 3 Institute for Microbiology, University of Veterinary Medicine Hannover, 30173 Hannover, Germany; peter.valentin@tiho-hannover.de; 4 Institute for Bacteriology and Mycology, Centre of Infectious Diseases, College of Veterinary Medicine, University Leipzig, 04103 Leipzig, Germany

**Keywords:** *Streptococcus suis*, stomach content, pig, oro-gastrointestinal infection

## Abstract

*Streptococcus* (*S.*) *suis* translocates across the intestinal barrier of piglets after intraintestinal application. Based on these findings, an oro-gastrointestinal infection route has been proposed. Thus, the objective of this study was to investigate the survival of *S. suis* in the porcine stomach. Whereas surviving bacteria of *S. suis* serotypes 2 and 9 were not detectable after 60 min of incubation in stomach contents with a comparatively high gastric pH of 5 due to feeding of fine pellets, the number of *Salmonella* Derby bacteria increased under these conditions. Further experiments confirmed the clearance of *S. suis* serotypes 2 and 9 within 30 min in stomach contents with a pH of 4.7 independently of the bacterial growth phase. Finally, an oral infection experiment was conducted, feeding each of 18 piglets a diet mixed with 10^10^ CFU of *S. suis* serotype 2 or 9. Thorough bacteriological screenings of various mesenteric-intestinal lymph nodes and internal organs after different times of exposure did not lead to any detection of the orally applied challenge strains. In conclusion, the porcine stomach constitutes a very efficient barrier against oro-gastrointenstinal *S. suis* infections. Conditions leading to the passage of *S. suis* through the stomach remain to be identified.

## 1. Introduction

Pigs are the natural host of *Streptococcus (S.) suis* and different mucosal surfaces are colonized by *S. suis* in many healthy pigs. However, virulent *S. suis* strains might cross different barriers of the host tissue and cause pathologies such as meningitis, arthritis and endocarditis [1,2]. Different serotypes have been identified in *S. suis* and differences in virulence among strains of different serotypes and even within serotype 2 are well known [3,4,5]. Serotypes 2 and 9 are very important in Europe and in China [6,7,8]. *S. suis* serotype 2 is also an important zoonotic pathogen [9]. Humans working with pigs or pork are at particular risk for *S. suis* infections [10]. In Vietnam, Thailand and Hong Kong, *S. suis* is one of the three most important bacterial agents causing meningitis in adult human beings [11,12]. Importantly, it has been shown that consumption of raw or undercooked meat is a major risk factor of this zoonosis in Vietnam [13].

The tonsils are considered to be an important entry site for invasive *S. suis* strains [2]. Recently it has been demonstrated that the swine influenza virus infection of respiratory epithelial cells promotes adhesion and invasion of *S. suis* serotype 2, suggesting that influenza virus–infected respiratory epithelium is also an important port of entry [14,15]. Interestingly, *S. suis* serotype 2 has also been demonstrated to reach mesenteric-intestinal lymph nodes of pigs after intestinal application or after oral application of gastric-acid fast capsules containing the bacteria [16,17]. These studies demonstrate that *S. suis* might cross the intestinal epithelial barrier but leave unanswered how *S. suis* might survive passage through the stomach. Thus, it is still not clear whether *S. suis* might also frequently cause oro-gastrointestinal infections in pigs as proposed.

*Salmonella enterica* is an oro-gastrointestinal pathogen causing different diseases in various animals. Pigs are very important carriers of zoonotic *Salmonella enterica*. Different studies have demonstrated that feed has a strong impact on the survival of salmonellae in the porcine stomach. A high water-binding capacity, high concentrations of organic acids and a pH below 4 in the stomach caused by coarsely ground meal feeding are associated with a prominent reduction of live salmonellae [18,19,20]. On the other hand, uptake of finely ground feed leading to a gastric pH close to 5 and a separation of the stomach contents into a sediment and liquid phase allows salmonellae to survive and infect new hosts.

Salmonellae respond to acid shock through a complex adaptation mechanism called the acid tolerance response, which includes the synthesis of over 50 acid shock proteins [21,22]. However, the low pH of the gastric milieu is not the sole bactericidal mechanism in the porcine stomach [22]. Notably, bacterial adaptation to the gastric milieu is similar but not identical to the acid shock response. This has been shown for *Campylobacter jejuni* which exhibits a distinct gene expression in the porcine stomach including the downregulation of ribosomal proteins and the upregulation of heat-shock and nitrosative stress proteins [23]. *S. suis* expresses an arginine deiminase system confering protection against acidity in the presence of high arginine concentrations [24], but the relevance of this finding for putative oro-gastrointenstinal infections and survival in the stomach is not known.

In this study we evaluated the survival of *S. suis* in the stomach contents of differently fed growing piglets as well as in conventional compound feed and conducted an oral infection experiment to study the role of oro-gastrointestinal infection in piglets. 

## 2. Results

### 2.1. Evaluation of Survival of S. suis in Porcine Stomach Contents ex Vivo

**Figure 1:**
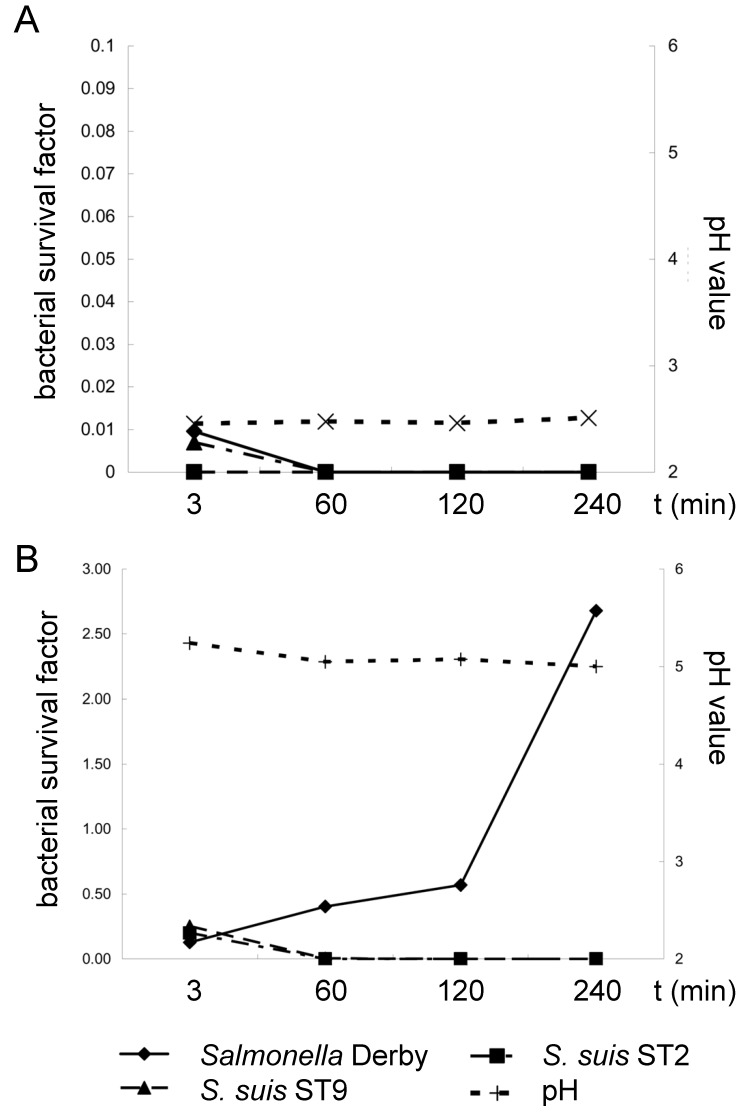
Mean survival factors of *S. suis* ST 2 strain 10, ST 9 strain A3286/94 and *Salmonella* Derby A147/85 as well as pH values in stomach contents ex vivo of piglets fed either a finely ground and pelleted (**A**) (*n* = 5) or coarsely ground meal diet (**B**) (*n* = 5). Stomach contents were mixed with bacteria and incubated for the indicated time points in air-tight sealed bags at 37 °C in a water bath. Standard deviations (SDs) are not included for reasons of clarity. At *t* = 3 min SDs were 0.192, 0.267 and 0.049 for *S. suis* ST2, ST9 and *Salmonella* Derby in (A), respectively. All other SDs were below 0.02 except for the values in (A) for *Salmonella* Derby at 60, 120 and 240 min with SD = 0.135; 0.191 and 1.32, respectively. The survival factor of *Salmonella* Derby was significantly higher at 240 min in comparison to the values at 120, 60 and 3 min (*p* < 0.05). Differences between survival factors at 120 and 3 min were also significant. The survival factor was calculated by dividing the specific bacterial content at a specific time point (CFU/g) by the inoculation dose.

This part of the study was designed to investigate the survival of *S. suis* serotypes 2 and 9 in porcine stomach contents ex vivo in comparison to the survival of *Salmonella enterica* (here Serovar Derby), a pathogen known to survive the gastric passage under feeding conditions, leading only to moderate acidic conditions in the stomach. Stomach contents were either collected from piglets fed coarse meal or fine pellets, leading to a mean gastric pH of 2.5 and 5.0, respectively (Figure 1). The content of culturable *S. suis* and *Salmonella* Derby bacteria were determined 3, 60, 120 and 240 min after mixing with the stomach contents. *S. suis* serotypes 2 and 9 showed mean survival/detection rates of 0.20 (±0.20) and 0.25 (±0.27) 3 min after mixing with the stomach contents of piglets fed fine pellets. The survival/detection rates were calculated by dividing the specific bacterial content determined for a specific time point by the inoculum used for this sample (inoculum in relation to the mass of the respective stomach contents). Of note, determination of the specific bacterial content of the *S. suis* serotypes was not hampered by the bacteria present in the stomach contents, as other typical α-hemolytic streptococci were not recorded and the selective media did not show overgrowth. After incubation of the mixed stomach contents for 60, 120 and 240 min at 37 °C, *S. suis* was not detectable anymore. In contrast, *Salmonella* Derby proliferated under these conditions, leading to a survival rate of 2.6 ± 1.3 after 240 min, significantly higher than the survival rates at 120 min and 60 min (Figure 1B). Three minutes after the mixing of bacteria with stomach contents with a pH below 3 of piglets fed coarse meal, the survival/detection rates of *S. suis* and *Salmonella* Derby were below 0.1. Incubation of these mixtures at 37 °C for 60 min or longer resulted in negative results for *S. suis* and *Salmonella* Derby (Figure 1B).

### 2.2. The Influence of the Growth Phase on the Survival of S. suis in Porcine Stomach Contents

**Figure 2:**
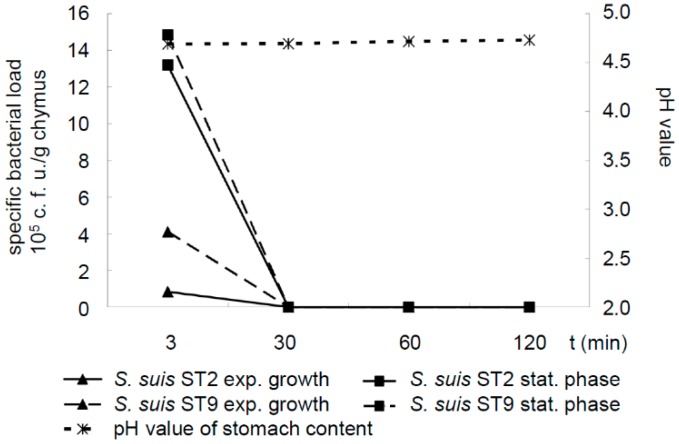
Mean specific bacterial loads of *S. suis* serotype 2 strain 10 and serotype 9 strain A3286/94 grown either to exponential (exp., OD_600_ = 0.6) or to stationary phase (stat., OD_600_ = 1.2) as well as pH values in stomach contents ex vivo of piglets (*n* = 6) fed a finely ground and pelleted diet. Stomach contents were mixed and incubated for the indicated time points in air-tight sealed bags at 37 °C in a water bath. At *t* = 3 min SDs were 1.3, 24.6, 4.8 and 19.0 for *S. suis* serotype (ST) 2 (exp. phase), ST2 (stat. phase), ST9 (exp. Phase) and ST9 (stat. phase), respectively. All other SDs were below 0.01. The differences of the specific bacterial loads at t = 3 min compared to the respective values of any other time point of analysis were significant (*p* < 0.05).

The arginine deiminase system, which is highly expressed in the stationary but not in the exponential growth phase, has been shown to protect *S. suis* against low pH [25,26]. Thus, we investigated the survival of the *S. suis* serotype 2 and 9 strains grown either to the exponential or the stationary phase in stomach contents with a moderate pH around 4.7 due to feeding fine pellets. However, live *S. suis* bacteria were only detectable 3 min but not 30, 60 or 120 min after 37 °C incubation in these stomach contents, independently of the growth phase chosen for collecting streptococci. In conclusion, we did not identify conditions leading to the detectable survival of *S. suis* in porcine stomach contents for at least 30 min incubation (Figure 2).

### 2.3. Survival of S. suis in Compound Pig Feed

As *S. suis* is a common bacterium in the oral and nasal cavity of piglets, contamination of feed with *S. suis* is a likely process during feeding. We monitored the survival of *S. suis* in two kinds of compound feed in comparison to the survival of *Salmonella* Derby, as salmonellae are known to be transmitted via contaminated feed. Though feed might be contaminated with the two pathogens by different mechanisms, a comparison with salmonellae was included to demonstrate bacterial survival in the stomach contents under specific conditions. 

**Figure 3:**
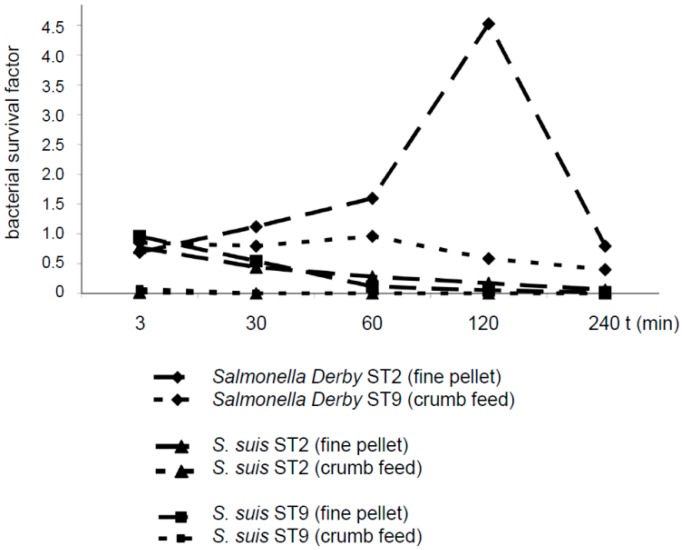
Survival factors of *Salmonella* Derby A147/85, *S. suis* serotype (ST) 2 strain 10 and *S. suis* ST 9 strain A3286/94 in compound feed (either in fine pellets without formic acid or as crumb feed including formic acid). Feeds were mixed either with 1.9 × 10^7^ CFU *Salmonella* Derby A147/85, 7.5 × 10^8^ CFU *S. suis* ST 2 strain 10 or 6.8 × 10^8^ CFU *S. suis* ST 9 strain A3286/94 per g feed and incubated for the indicated time points at room temperature (20 to 24 °C). The survival factor was calculated by dividing the specific bacterial content at a specific time point (CFU/g) by the inoculation dose.

*S. suis* serotypes 2 and 9 were reduced in number in diet 1 (fine pellet not including formic acid) after an incubation period ranging from 30 to 240 min. After incubation in diet 2 (crumb feed including formic acid), neither *S. suis* serotypes 2 or 9 were detectable at any time point after mixing (Figure 3). In contrast, *Salmonella* Derby increased in number in diet 1, reaching a survival rate of 4.5 after 120 min. In diet 2, *Salmonella* Derby showed a survival factor close to 1 after incubation for the first 60 min, followed by a numerical reduction to a survival factor of approximately 0.4 at 240 min.

### 2.4. Determination of Acid Sensitivity of S. suis

We considered the low pH in the stomach contents and the feed to be bactericidal to *S. suis*. To address this further, formic acid was added to Todd-Hewitt broth (THB) in different concentrations to obtain pH values of 4.0, 4.3 and 5.3, and streptococci grown to exponential or stationary phase were added to these solutions. *S. suis* serotypes 2 and 9 were completely killed in THB plus formic acid with pH 4.0 within 1 h. At pH 4.3, *S. suis* serotypes 2 and 9 grown to the stationary but not to the exponential growth phase survived with low numbers of about 10^3^ CFU/mL after 1 h incubation. Interestingly, *S. suis* serotypes 2 and 9 grown to stationary phase survived for 1 h at a pH of 5.3 in THB without a detectable reduction in the bacterial numbers. 

### 2.5. Oral Infection Experiment of Piglets with S. suis

The in vitro results of this study indicated the clearance of *S. suis* serotypes 2 and 9 in different stomach contents, which was most likely related at least in part to the low pH. Because of data obtained in other studies suggesting oro-gastrointestinal infection, we nevertheless conducted an oral infection experiment with piglets designed to test whether *S. suis* causes intestinal infection after passage through the stomach. The bacteria were mixed with individual portions of feed to best mimic putative oro-gastrointestinal infection in the field. None of the piglets showed specific clinical signs of *S. suis* infection such as lameness or central nervous system dysfunction after oral application of 1 × 10^10^ CFU *S. suis* serotypes 2 or 9, a dose almost 10-fold higher than the dose used for inducing mortality after intranasal application in serotype 2 infection experiments. However, numerous piglets in each group including the control group demonstrated elevated body temperatures at different time points. The white blood cell count on days 5, 8, 11 and 15 post *S. suis* application revealed values below 22 × 10^9^/l except for one animal in the serotype 2– and the serotype 9–infected group. The reason for the elevated body temperatures is unclear, but an association with *S. suis* infection is less likely, as control piglets (kept in a separate unit) were also affected and the high body temperature was not associated with leucocytosis or the detection of fibrinosuppurative lesions (see below).

As the incubation time of oro-gastrointenstinal *S. suis* infection is completely unknown, piglets were sacrificed at different time points post oral *S. suis* application (5, 8, 11, 15 dpi). As our aim was to demonstrate oro-gastrointestinal infection, we investigated numerous intestinal lymph nodes (*Lnn. jejunales*, *ileocolici* and *colici*) in addition to various internal organs bacteriologically and histologically. Neither *S. suis* serotypes 2 or 9 were detected in any of the internal organs or in any of the investigated intestinal lymph nodes. Furthermore, none of the piglets which had received feed mixed with either *S. suis* serotype 2 or serotype 9 showed a fibrinous or suppurative lesion in any of the investigated tissues including various mesenteric intestinal lymph nodes with the exception of one piglet with a focal suppurative jejunal lymphadenitis. The latter was, however, not positive for the challenge strain and other pathogenic bacteria were also not detected in this lymph node. It is worth noting that the animal experiment was designed to reveal intestinal infection leading at least to infection of the intestinal lymph nodes. Colonization or passage of the intestinal tract was not investigated. In conclusion, the oral infection experiment did not indicate intestinal infection after uptake of compound feed mixed with 10^10^ CFU *S. suis* serotype 2 or 9. 

## 3. Discussion

The stomach acts as a barrier preventing harmful bacteria from entering and proliferating in the lower part of the gastrointestinal tract. One important defence mechanism of this barrier is its low pH. In this study we demonstrated that a virulent *S. suis* serotype 2 and a virulent serotype 9 strain were killed in the stomach contents of pigs fed different diets, including a finely ground and pelleted diet leading to a comparably high pH of 5 in the stomach. The latter allowed salmonellae, a well-known oro-gastrointestinal pathogen, not only to survive but even to proliferate, indicating that salmonellae pass the stomach efficiently under these conditions in contrast to *S. suis*. Furthermore, we did not detect our challenge strains after oral application in any internal organ or mesenteric intestinal lymph node. In contrast, Ferrando et al. [16] recorded *S. suis* serotypes 2 and 9 after oral application in mesenteric intestinal lymph nodes and translocation across a porcine intestinal epithelial cell barrier in vitro, indicating that *S. suis* might pass the intestinal epithelial barrier. However, the authors applied *S. suis* in gastric acid–resistant capsules. Thus, intestinal infection by *S. suis* might occur once the pathogen has passed the stomach, but the conditions leading to sufficient passage through the stomach are unclear. It appears unlikely that oro-gastrointestinal infection constitutes a main infection route in growing piglets fed diets similar to the ones used in this study, as *S. suis* did not survive in compound feed with commonly used additives such as formic acid, and was rapidly killed in stomach contents of differently fed growing piglets and infection was not observed after administration of very high oral doses. However, the putative passage of *S. suis* through the stomach during the suckling period merits further investigation.

The findings of this study are surprising with regard to an earlier publication describing *S. suis* as the predominant bacterial species in the stomach of 21-day-old weaning piglets [27]. Though this difference might be related to the change in diet during weaning, it is also worth mentioning that the authors used a real-time PCR assay for *S. suis* detection. They did not confirm live *S. suis* bacteria by culture, which is very important as porcine saliva contains *S. suis* in high numbers. Thus, their results do not confirm the passage of live *S. suis* through the porcine stomach.

*S. suis* is thought to colonize the intestinal mucosa of pigs efficiently. This hypothesis is mainly based on the detection of the 16S rRNA gene in intestinal contents [28,29]. However, to the best of our knowledge, the role of the porcine intestinal tract as a putative reservoir and site of infection for important *S. suis* pathotypes has not been investigated at all. Interestingly, Devriese et al. [30] showed that most of the intestinal *S. suis* bacteria belonged to a beta-glucuronidase–negative biotype, which is only rarely detected in invasive *S. suis* infections. It is important to characterize putative *S. suis* isolates of the intestinal tract in future studies using state-of-the-art phenotyping and genotyping.

Based on our results, *S. suis* serotypes 2 and 9 did not survive in stomach contents with a pH = 5 for 60 min, though stable numbers of viable, stationary phase bacteria were recorded in THB with a pH = 5. Thus, porcine gastric fluid exhibited further bactericidal effects on *S. suis* in addition to a low pH. This has also been shown for *Salmonella* Typhimurium mutants [22] and *Listeria monocytogenes* [31].

Interestingly, Grüning et al. [26] demonstrated the survival of *S. suis* at a pH of 4 in the presence of 25 mM l-arginine. This phenotype was reduced in an isogenic mutant of the *arcA* gene encoding the arginine deiminase. Thus, the arginine deiminase system might be important to cope with the low pH in the stomach. However, free arginine is very limited in the diets of pigs [32,33]. As free l-arginine might reach higher concentrations after the uptake of food of animal origin, *S. suis* might still pass through the human stomach in high numbers after eating “high-risk dishes”, which were identified in a case-control study conducted in Vietnam [13]. Thus, the results of this study do not at all exclude oro-gastrointestinal infection as an important infection route in humans eating undercooked meat, blood and intestine of *S. suis* ST2–infected pigs in Vietnam and other countries. However, colonization of the human intestinal tract by *S. suis* seems to be uncommon, as rectal samples of more than 1500 healthy individuals were negative in a PCR for *S. suis* [13]. 

The presented data suggests that the gastric passage of live *S. suis* bacteria is an uncommon process in growing piglets, the age class most often and most severely affected by *S. suis* diseases. This does not rule out that *S. suis* diseases in growing piglets develop after intestinal translocation of bacteria that have colonized the intestinal tract of these piglets in the previous weeks and that have passed through the stomach under very different conditions in the neonatal, suckling or weaning periods. Thus, it is important to conduct further studies on putative oro-gastrointestinal *S. suis* infections in piglets. This should include experimental designs to identify conditions leading to passage of the stomach, which is a very important barrier against *S. suis* infection, as shown in this study.

## 4. Materials and Methods

### 4.1. Bacteria

The *S. suis* reference strains 10 and A3286/94 were used in this study and are referred to as serotype 2 and 9 strain, respectively. Strain 10 is an MRP+ EF+ SLY+ serotype 2 strain which has been used successfully by different groups to induce disease experimentally in either intranasal or intravenous application models with piglets [34,35,36,37,38,39]. The MRP* SLY+ serotype 9 *S. suis* strain A3286/94 caused disease after intravenous injection but only subclinical pathological findings after intranasal application [40]. Cultivation of *S. suis* was conducted on Columbia agar with 6% sheep blood supplemented in appropriate cases with StaphStrep selective Supplement or in Todd-Hewitt broth (THB, all Oxoid, Wesel, Germany) at 37 °C for 24 h.

The *Salmonella enterica ssp. enterica* Serovar Derby (*Salmonella* Derby) strain A147/85 (O 1,4, 12; H:f,g) was cultivated on Columbia agar with 6% sheep blood, on Brilliance^TM^Salmonella agar, on Brilliant Green agar (all Oxoid, Wesel, Germany) or in lysogeny broth as appropriate.

### 4.2. Virulence-Associated Gene Profiling of Putative S. suis Isolates by PCR

A previously described multiplex (MP) PCR was used to detect the virulence-associated genes *mrp*, *epf*, *sly*, *gdh*, *arc*A, *cps*1J, *cps*2J, *cps*7H and *cps*9H in *S. suis* isolates [8].

### 4.3. Determination of Bacterial Survival in Porcine Stomach Contents ex Vivo

Stomach contents of the gastric fundus region were collected 6 h postprandial and directly after killing from male growing piglets (*n* = 5 per feed) with a body weight of 33.3 ± 6.0 kg. These piglets were from an SPF herd known to be free of salmonellae and *mrp*+ *epf*+ *sly*+ *cps*2 as well as *cps*9 *S. suis* strains. They were either fed a finely ground and pelleted (“fine pellets”; *n* = 5) or coarsely ground meal diet (“coarse meal”; *n* = 5) with a dry matter content of 914 g/kg and 884 g/kg, respectively. The particle size distribution after wet sieve analysis of fine pellets showed 5% larger than 1 mm and 54% smaller than 0.2 mm resulting in a geometric mean diameter (GMD) of 217 µm. Corresponding parameters for the coarse meal were 53% larger 1 mm and 26% smaller than 0.2 mm and 671 µm GMD. The energy contents of the fine pellets and coarse meal were 13.9 MJ/kg and 13.5 MJ/kg, respectively. The different stomach contents resulting from the feeding of these diets were used for comparative analysis of survival of *S. suis* serotypes 2 and 9 as well as *Salmonella* Derby.

For analysis of survival of *S. suis* serotypes 2 and 9 grown to exponential versus stationary phase contents of the gastric fundus region were collected 6 h postprandial from 6 piglets with a body weight of 22.3 ± 2.7 kg fed fine pellets with 42.4% of particles smaller than 0.2 mm and 292 µm GMD. After necropsy of piglets stomach contents were transferred to sterile plastic containers and kept at −20 °C until use.

For determination of bacterial survival 10 g stomach content was mixed with 1 mL of a bacteria-PBS suspension containing a calculated specific bacterial load of 2 × 10^8^ CFU of either *Salmonella* Derby A147/85 (grown to OD_600_ = 0.6), *S. suis* serotype 2 strain 10 or *S. suis* serotype 9 strain A3286/94 (both grown to OD_600_ = 0.8) or as negative control PBS without bacteria. Accompanying bacteria of the stomach contents did not overgrow the Columbia blood and StaphStrep selective agar plates and did not include similar α-haemolytic streptococci. All typical α-haemolytic streptococci were screened in the MP-PCR for virulence-associated genes to confirm the genotype used for inoculation. This was conducted for each of the indicated time points of analysis in a separate air tight sealed bag. Bags were incubated at 37 °C in a shaking water bath after mixing. The pH in the stomach content was measured at every sampling. A 10-fold dilution series in PBS was made for determination of the specific bacterial load. The first dilution was homogenized in an air tight sealed bag using a stomacher (Stomacher 400 Circulator, Fa. Seward, Worthing, West Sussex, UK). Dilutions were plated onto selective media for streptococci and salmonellae as well as on Columbia blood agar as specified above. The survival factor was calculated by dividing the specific bacterial content at a specific time point (CFU/g) by the inoculation dose. The second experiment investigating survival of *S. suis* grown to different ODs comparatively was conducted alike only that 1.8 × 10^8^ CFU of *S. suis* serotype 2 strain 10 or *S. suis* serotype 9 strain A3286/94 grown to OD_600_ = 0.6 or 1.2 (as indicated) were mixed with the stomach content.

### 4.4. Determination of Bacterial Survival in Pig Feed

Ten g of conventional compound feed (dry starter diet, either as fine pellet or as crumb feed) based on wheat, barley and soybean was mixed with 1 mL PBS solution containing 1.9 × 10^8^ CFU *Salmonella* Derby or 7.5 × 10^9^ CFU *S. suis* serotype 2 or 6.8 × 10^9^ *S. suis* serotype 9 and incubated for the indicated time points (3, 30, 60, 120 and 240 min) at room temperature. The compound feed in crumb form contained also 3.5 g formic acid/kg uS. The specific bacterial load was determined using the stomacher for homogenization and a 10-fold dilution series in PBS as described above. Quantification was possible as the accompanying bacteria of the feed did not overgrow the media used for cultivation and did not include α-haemolytic streptococci. The *S. suis* genotypes were confirmed in the MP-PCR for virulence-associated genes.

### 4.5. Determination of Survival of S. suis in Culture Medium after Addition of Formic Acid

Formic acid (98%–100%, Sigma-Aldrich-Chemie GmbH, Taufkirchen) was added at concentrations of 3.5, 2.4 and 1.2 g/kg to 10 ml THB immediately after addition of 1 × 10^8^ CFU *S. suis* serotypes 2 or 9 grown as indicated to OD_600_ = 0.6 or 1.2. This led to a pH of 4.0, 4.3 and 5.3, respectively. THB-medium without formic acid was included as positive control.

For addition of defined numbers of bacteria the *S. suis* strain was grown to OD_600_ = 0.6 and 1.2. Aliquots of these cultures were frozen via liquid nitrogen after addition of 10% glycerol. The specific bacterial content of these aliquots was determined using serial dilutions.

### 4.6. Oral S. suis Infection of Piglets

The piglets used in this study were cared for in accordance with the principles outlined in the European Convention for the Protection of Vertebrate Animals Used for Experimental and Other Scientific Purposes and the German Animal Protection Law. The animal experiment was approved by the Lower Saxonian State Office for Consumer Protection and Food Safety (permit no. 33.14-42502-04-12/0803, LAVES). The 27 piglets used in this study were from a herd that is free of *cps*7, *cps*9 and *mrp*+ *epf*+ *sly*+ *cps*2 *S. suis* strains. After weaning the piglets were trained to feed from individual plastic containers allowing application of a distinct oral dosage of bacteria through feeding. Commercially available compound feed in crumb form (deuka primo pro gran, Deutsche Tierernährung Cremer GmbH & Co. KG, Bramsche, Germany) with 15.5 MJ ME/kg feed including 3.5 g formic acid/kg uS was used for restricted feeding after weaning. The feed’s particle size distribution was 28% larger than 1 mm and 42% smaller than 0.2 mm. After 18 h abrosia each piglet received 50 g feed prior to application of the infection dose to increase the gastric pH. After a 5 min pause piglets received 10 g feed supplemented with 5 ml of a PBS suspension including either 10^10^ CFU *S. suis* serotype 2, 10^10^ CFU *S. suis* serotype 9 or no added bacteria (for each *n* = 9 piglets). The health of the piglets was closely monitored including measurement of body temperature, evaluation of feeding, breathing and movement of animals as well as fecal consistency every 8 h. A body temperature above 40.0 °C was considered elevated. On days 1, 5, 8, 11 and 15 with regard to *S. suis* application blood samples were taken from all living piglets for hematological screening. White blood cells were counted using a hemocytometer chamber. Leukocytes were differentiated by Wright stained blood smears.

Two piglets per group (serotype 2, serotype 9 and control) were sacrificed on days 5, 8 and 15 post *S. suis* application. On day 11 three piglets per group were killed. Necropsy was conducted with every animal including comprehensive sampling for bacteriological and histological examinations as described [34,35]. In addition to various samples of internal organs taken in all our infection experiments numerous *Lnn. jejunales*, *Lnn. ileocaecales* and *Lnn. colici* were collected and investigated bacteriologically and histologically. For bacteriological analysis the lymph nodes were cut in many sections. The cut surfaces were slightly pressed against Columbia blood and StaphStrep selective agar plates. The plates were streaked for single colonies. All α-hemolytic streptococci detected in internal organs or mesenterial intestinal lymph nodes were screened in the MP PCR for profiling of virulence-associated factors. 

### 4.7. Statistics

Statistical analysis was performed by SAS 9.3 for Windows using PROC MEANS or the Wilcoxon test for differences between time points and student’s *t*-test (PROC GLM) for those between groups or bacteria. Values are expressed as mean ± standard deviation (SD). Probabilities lower than 0.05 were considered significant.
